# Promoter Hypermethylation Profiling Identifies Subtypes of Head and Neck Cancer with Distinct Viral, Environmental, Genetic and Survival Characteristics

**DOI:** 10.1371/journal.pone.0129808

**Published:** 2015-06-22

**Authors:** Javed Hussain Choudhury, Sankar Kumar Ghosh

**Affiliations:** Molecular Medicine Laboratory, Department of Biotechnology,Assam University, Silchar, Pin-788011, Assam, India; Peking University Cancer Hospital and Institute, CHINA

## Abstract

**Background:**

Epigenetic and genetic alteration plays a major role to the development of head and neck squamous cell carcinoma (HNSCC). Consumption of tobacco (smoking/chewing) and human papilloma virus (HPV) are also associated with an increase the risk of HNSCC. Promoter hypermethylation of the tumor suppression genes is related with transcriptional inactivation and loss of gene expression. We investigated epigenetic alteration (promoter methylation of tumor-related genes/loci) in tumor tissues in the context of genetic alteration, viral infection, and tobacco exposure and survival status.

**Methodology:**

The study included 116 tissue samples (71 tumor and 45 normal tissues) from the Northeast Indian population. Methylation specific polymerase chain reaction (MSP) was used to determine the methylation status of 10 tumor-related genes/loci (*p16*, *DAPK*, *RASSF1*, *BRAC1*, *GSTP1*, *ECAD*, *MLH1*, *MINT1*, *MINT2* and *MINT31*). Polymorphisms of *CYP1A1*, *GST* (*M1* & *T1*), *XRCC1*and *XRCC2* genes were studied by using polymerase chain reaction-restriction fragment length polymorphism (PCR-RFLP) and multiplex-PCR respectively.

**Principal Findings:**

Unsupervised hierarchical clustering analysis based on methylation pattern had identified two tumor clusters, which significantly differ by CpG island methylator phenotype (CIMP), tobacco, *GSTM1*, *CYP1A1*, HPV and survival status. Analyzing methylation of genes/loci individually, we have found significant higher methylation of *DAPK*, *RASSF1*, *p16* and *MINT31*genes (*P = *0.031, 0.013, 0.031 and 0.015 respectively) in HPV (+) cases compared to HPV (-). Furthermore, a CIMP-high and Cluster-1 characteristic was also associated with poor survival.

**Conclusions:**

Promoter methylation profiles reflecting a correlation with tobacco, HPV, survival status and genetic alteration and may act as a marker to determine subtypes and patient outcome in HNSCC.

## Introduction

Globally, head and neck cancer is the fifth most common cancer and accounts for more than 550,000 new cases each year [1]. Consumptions of tobacco (smoking and chewing), alcohol and HPV infection are well-known risk factors toward the development and progression of head and neck squamous cell carcinoma (HNSCC) [2, 3]. In India, HNSCC has profound smoking, betel quid and tobacco chewing profile and comparatively poor survival [4–6]. However, HPV positive (+) HNSCC had distinct risk profile and associated with a better survival compared to HPV negative (-) HNSCC [7]. Genetic alteration such as mutations and genetic polymorphisms are also play a key mechanism in HNSCC [8–11]. In addition, epigenetic modification (variation of gene expression without affecting primary sequence of the DNA) can alter the expression of key tumor-related genes and thus considered as a crucial player to the development of various cancers [12–14].

Hypermethylation of CpG islands in the promoter region of genes (those involved in cell cycle regulation, apoptosis, DNA damage repair and detoxification pathway) are associated with cancer progression and development. Thus, aberrant methylation of CpG islands is one of the epigenetic modifications promising immense potential molecular biomarker for prediction and detection of a variety of cancers [15, 16]. CpG island methylator phenotype (CIMP) associated tumors are a distinct group defined by CpG–rich promoter hypermethylation in multiple genes and have a distinct epidemiology and molecular features [17–20]. The concept of CIMP was first proposed in colorectal cancer as a molecular marker [21], later it was also studied in other tumor types. However, the role of CIMP pathway in the tumorgenesis of HNSCC is still unknown. The major confront in studying and exploring CIMP associated tumors is to define specific methylated loci that should be used as CIMP panel. Aberrant methylation of the normally unmethylated CpG islands is associated with transcriptional inactivation and thus loss of gene expression. Recent investigations conducted on different tumor-related genes also shown differential methylation pattern in HNSCC [22–26]. To evaluate and screen CIMP status of cancers, Park etal [27] proposed a panel of genes consisting *of p16/CDKN2A*, *MINT1*, *MINT2*, *MINT31* and *MLH1* (referred as classical CIMP panel).The frequencies of hypermethylation in a panel of six genes (*ECAD*, *p16*, *DAPK*, *MGMT*, *RASSF1* and *TIMP3*) were found very high in head and neck cancer [28]. In another study, aberrant methylation of *MINT1* and *MINT31* was found to be associated with poor prognosis [29]. Earlier studies also reported that *p16* and *DAPK* aberrant methylation was associated with poor prognosis in oral cancers [30, 31]. Therefore, we proposed a CIMP panel of seven genes, including; *DAPK* (death-associated protein kinase), *RASSF1* (Ras association domain family-1), *BRCA1* (breast cancer 1), *MLH1* (mutL homology 1), *p16* (cyclin-dependent kinase inhibitor 2A), *ECAD* (epithelial cadherin), *GSTP1* (glutathione S-transferase pi-1), and three methylated loci such as *MINT1*, *MINT2* and *MINT31* (methylated in tumor 1, 2 and 31 respectively). Recent epigenetic studies on various cancers mainly focused on multigene approach, associations with HPV infection and clinicpathological data and with genetic alterations [32–34]. The HPV oncoproteins E6 and E7 are known to be associated with genomic instability by inactivating p53 and Rb tumor suppressor proteins of cell cycle pathway [35], apart from this, HPV also modulates aberrant DNA methylation of the host genome [36] and cause carcinogenic processes. The past few years, many studies had conducted to explain association of HNSCC with alternation of xenobiotic and DNA repair pathway genes as well as gene-gene and gene-environment interaction [37, 38]. Tahara et al. [39] showed genetic factors, related to DNA repair or xenobiotic pathways may have a role in CpG island hypermethylation-related gastric carcinogenesis. However, there were no such studies conducted focusing specific promoter methylation profile in HNSCC with a combination of genetic risk factors related to xenobiotics and DNA repair pathway. Thus, variation in promoter hypermethylation pattern of HNSCC based on habits, genetic alteration and HPV infection remains unclear.

To understand the underlying mechanisms of differences in patterns of tumor-specific hypermethylation, we have analyzed the aberrant promoter methylation profile of HNSCC using seven important tumor-related pathway genes, including *DAPK*, *RASSF1* (apoptosis pathway), *BRCA1*, *MLH1* (DNA repair pathway), *p16* (cell-cycle pathway), *ECAD* (cell-cell adhesion), *GSTP1* (Xenobiotic pathway) and three methylated loci (*MINT1*, *MINT2* and *MINT31*) in the high cancer incidence zone of Northeast India. To the best of our knowledge, we are the first to explore; the correlation of CIMP characteristics with genetic (polymorphisms of *GSTM1*, *GSTT1*, *CYP1A1*, *XRCC1* and *XRCC2* genes) and environmental factors (smoking, betel quid and tobacco chewing) and also with HPV and survival status of HNSCC patients. Furthermore, we performed hierarchical cluster analysis to identify distinct subsets of HNSCC based on the promoter methylation profile.

## Materials and Methods

### Collection of HNSCC tissues

In the present study, we examined 116 tissue specimens, including 71 tumor samples of HNSCC and 45 adjacent normal tissues, collected from different hospitals of Northeast India from 2009–2013. Patients gave their written informed consent before collection of the samples. Basic demographic data like age, gender, tobacco (smoking/chewing) consumption, food habits etc. were collected using a standard questionnaire.

### Ethics Statement

Collection, consent form and analysis of tissue samples were approved by Institutional Ethical Committee (IEC), Assam University, Silchar, Assam, India.

### DNA extraction

Genomic DNA was extracted from biopsy samples, surgically excised cancer tissues, and adjacent normal tissues using standard phenol/chloroform extraction protocol and also by Bioline Isolate Genomic DNA minikit (Bioline, UK) following manufacturer’s instructions. The extracted DNA was then dissolved in TE (10 mM Tris-HCl pH 8.0, 1 mM EDTA) buffer and stored at -80°C for further analysis [40].

### HPV detection

The tissue samples were screened using set of consensus primers My9/My11 for amplifying HPV L1 gene fragments, that can detect high-risk strains of HPV [32]. Amplification was carried out on 20 μl reaction mixtures containing 2X Biomix (Bioline, UK), forward and reverse primers, 2 μl sample and nuclease free water. The PCR reaction mixture was subjected to initial denaturation at 94°C for 5 min, followed by 35 cycles at 94°C for 45s, 47°C for 1min and 72°C for 1 min. The final extension was done at 72°C for 10 min. All possible precautions, including negative controls were taken to minimize cross contamination. The PCR products were observed in 2% agarose gel with ethidium bromide staining.

### Genetic polymorphisms of carcinogen metabolizing (*GSTM1*, *GSTT1* and *CYP1A1*) and DNA repairs (*XRCC1* and *XRCC2*) genes

The polymorphisms of *CYP1A1* (T3801C), *XRCC1* (Arg399Gln) and *XRCC2* (Arg188His) genes were analyzed by polymerase chain reaction-restriction fragment length polymorphism (PCR-RFLP) method. The polymorphic site of *CYP1A1*, *XRCC1* and *XRCC2* was amplified using published forward and reverse primers [37, 41] in 20 μl PCR reactions. Each PCR reaction mixture contains 10–100 ng genomic DNA, 20 pmoles of each primer, 10X reaction buffer, dNTP mix, *Pfu* DNA polymerase, MgCl_2_ and nuclease free water (NFW). The PCR reaction mixture was set with an initial denaturation at 94^°^C for 5 min, followed by 35 cycles of 94^°^C for denaturation for 45s, 62^°^C/ 58^°^C /60^°^C (for *CYP1A1*, *XRCC1* and *XRCC2* respectively) for 45s for primer annealing and extension at 72^°^C for 1 min. The final extension was done at 72^°^C for 10 min. PCR products of *CYP1A1*, *XRCC1* and *XRCC2* were digested separately with the restriction enzyme *Msp1*, *HpaII* and *HphI* enzymes (New England BioLabs, USA) respectively. The digested products were then resolved on 3% agarose gel to assess the size of the PCR–RFLP products [37]. Genotyping of the *GSTM1* and *GSTT1* was done by multiplex PCR, using *CYP1A1* gene as an internal control, in a total volume of 10 μl reaction mixture of 2X Biomix (Bioline, UK) and 10 pmole of each of the forward (F) and reverse (R) primers (**S2 Table**). The PCR products were electrophoresed in 1.5% agarose gel stained with ethidium bromide.

### Promoter hypermethylation analysis and assessment of CIMP-status

Promoter methylation status of tumor-related genes (*RASSF1*, *DAPK*, *ECAD*, *BRCA1*, *MLH1*, *p16* and *GSTP1*) and three methylated loci (*MINT1*, *MINT2*, and *MINT31*) were analyzed using Methylation Specific PCR (MSP) primers (**S2 Table**). For MSP assay, DNA samples were subjected to modification using Imprint1 DNA Modification kit (Sigma–Aldrich, St. Louis, MO), following instructions as described by manufacturer’s [18]. In this procedure, DNA denaturation and bisulfite modification are carried out simultaneously. Bisulfite reacts with single-stranded DNA to deaminate the cytosine (C) and transforms unmethylated cytosine (C) to uracil (U) and leaves 5-methyl cytosine unchanged and thus creates different sequences for methylated and unmethylated DNA. Then we have used two different sets of primers for each gene, one specific set of primers for methylated DNA and the other for unmetyhlated DNA. We also used DNA from peripheral blood lymphocytes of healthy individuals without HPV infection, as negative control, and DNA from peripheral blood lymphocytes treated with SssI methyltranferase was used as positive control (for methylated DNA). All the PCR reaction was performed in gradient thermal cycler (Applied Biosystems, Inc, CA, USA) and the amplified products for methylated and unmethylated DNA were run side by side on agarose gel for comparison. In this study, CIMP panel was classified into three groups: CIMP-high (at least 5 genes/loci methylated out of 10), CIMP-low (less 5/10 genes/loci methylated), and CIMP-negative (0/10 genes/loci methylated). This classification was done on the basis of the criteria previously used for CIMP status in other types of tumor [19, 42]. Methylation index (MI) was also calculated for each case via dividing the number of Methylated genes/loci by the total number of gene/loci under study.

### Survival analysis

Survival was calculated in months from the beginning of treatment to the month of deathusing Kaplan–Meier survival curves in SPSS software, version 18 (Windows). Deaths due to diseases/complication other than cancer were expelled from the study. The association between different characteristics (HPV, CIMP and cluster) and the event of death was analyzed using Log–rank (Mantel–Cox) tests.

### Statistical analysis

Statistical analysis was performed using SPSS software version 18 and two-sided *P*-value 0.05 (two-tailed) was considered statistically significant. We used the Wilcoxon rank-sum test to compare promoter methylation levels of HNSCC tumor samples and normal samples, which permit the comparison of two groups of independent samples [43]. The significant values were further adjusted for multiple testing by Bonferroni method (*P*-value multiplied by number of comparisons). Also, to strengthen the association between different factors and CIMP in HNSCC risk, *P*-values were calculated after adjusting confounding factors such as age, gender, HPV, smoking, betel-quid and tobacco chewing status as appropriate. Test for linear trend was also carried for multiple ordinal CIMP status. Unsupervised hierarchical clustering was done using JMP 12 software package of SAS, which identify subgroups among HNSCC patients based on promoter methylation frequency.

## Results

### Characteristics of patients

The clinicopathological data of the 71 studied HNSCC patients are summarized in **Table 1,** comprised 51 (71.8%) male and 20 (28.2%) females with an age range of 23–86 years (77.5% belong to the age group 45–86). Of the 71 HNSCC patients; 38 (53.5%) oral cancer (including cheek, base of the tongue, tongue, gingivam, and buccal mucosa) 16 (22.6%) had laryngeal cancer, 8 (11.2%) had pharyngeal cancer and 9 (12.7%) had other cancer types in head and neck region. According to TMN classification, the majority of patients had advanced stage (III/IV) (67.3%) at diagnosis. Among the HNSCC patients, smokers, betel quid chewers and tobacco chewers were 71.8%, 66.2% and 74.6% respectively.

**Table 1:** Clinicopathological data of HNSCC patients under study.

Parameters	Cases (N)	Percentage (%)
Gender		
Male	51 (71)	71.8
Female	20 (71)	28.2
Age		
≤45	16 (71)	22.5
>45	55 (71)	77.5
Smoking		
Non-smokers	20 (71)	28.2
Smokers	51 (71)	71.8
Betel quid chewing		
Non-chewers	24 (71)	33.8
Chewers	47 (71)	66.2
Tobacco chewing		
Non-chewers	18 (71)	25.4
Chewers	53 (71)	74.6
Stage		
Local (I/II)	17 (52)	32.7
Advanced (III/IV)	35 (52)	67.3
NA	19 (71)	
Tumor site		
Laryngeal	16 (71)	22.6
Pharyngeal	8 (71)	11.2
Oral	38 (71)	53.5
Base of tongue	8 (71)	11.2
Tongue	4 (71)	5.6
Cheek	16 (71)	22.6
Gingivam	4 (71)	5.6
Buccal mucosa	6 (71)	8.4
other	9 (71)	12.7

NA : not available

### Frequencies of promoter hypermethylation in tumor samples of HNSCC patients and normal tissues

**Fig 1:**
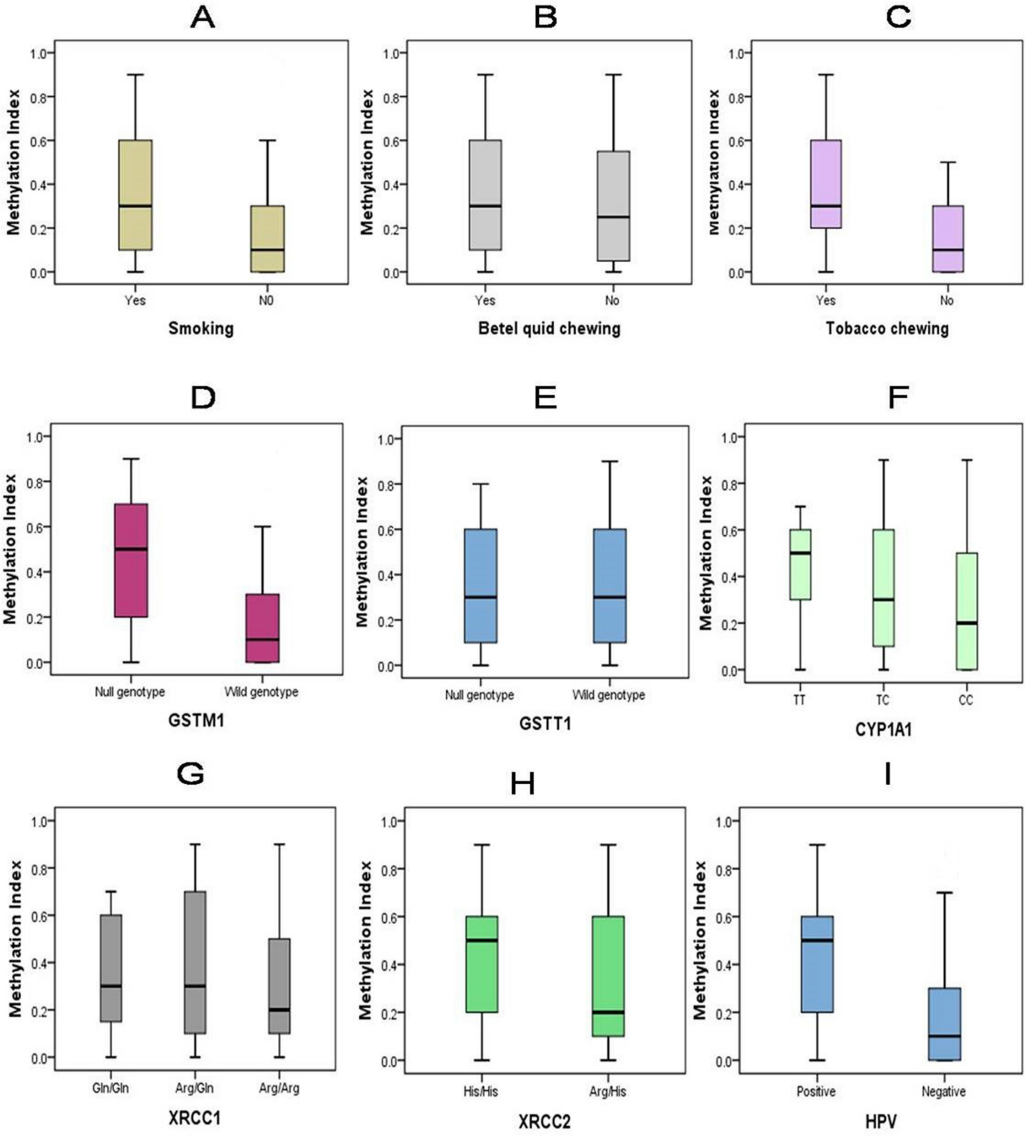
Methylation index (MI) stratified by genetic and habit related risk factors in HNSCC. Each box plot represents differential methylation index (MI) among smokers/chewers and non smokers/chewers or wild genotype vs. variant genotype or HPV (+) vs. HPV (-) HNSCC patients.

Promoter hypermethylation status of the *p16*, *DAPK*, *GSTP1*, *RASSF1*, *BRCA1*, *ECAD*, *MLH1*, *MINT1*, *MINT2* and *MINT31* genes of 71 HNSCC and 45 normal tissues samples was shown in **Table 2**. Tumor tissues had much higher genes/loci hypermethylation frequency compared to normal tissue samples (32.4% vs. 13.3% for *p16*, 29.6% vs. 11.1% for *DAPK*, 18.3% vs. 8.9% for *BARC1*, 31% vs. 15.6% for *GSTP1*, 32.4% vs. 8.9% for *ECAD*, 50.7% vs. 22.2% for *RASSF1*, 5.6% vs. 2.2% for *MLH1*, 43.7% vs. 13.3% for *MINT1*, 52.1% vs. 11.1% for *MINT2* and 46.5% vs. 17.8% for *MINT31*). However, significantly high level of hypermethylation was observed in *p16*, *DAPK*, *ECAD*, *RASSF1*, *MINT1*, *MINT2* and *MINT31* (*P* = 0.02, 0.02, 0.04, 0.02, 0.01, <0.01, and 0.01 respectively) in HNSCC tissues compared to normal tissue samples. The methylation index (MI) (ratio of the number of methylated promoters and total number of promoters under study) ranged from 0 to 0.9 of the 71 patients. Out of the 71 HNSCC patients 14 (19.7%) had 0 (zero) MI, 33 (46.5%) had MI of 0.1–0.5 and 24 (33.8%) patients had MI of 0.6–0.9. HNSCC patients with different habits and genetic profiles were found to exhibit differential methylation index (MI). Mean MI of tobacco chewers/smokers was higher than non chewers/smokers. Similarly, patients with *GSTM1* null, *CYP1A1* CC genotype and HPV (+) also shown higher methylation index (**Fig 1**).

**Table 2:** Frequency of methylation of follow genes in normal and tumor tissues of HNSCC patients.

Genes/loci	Frequency of methylation (%)		
	Tumor tissue	Normal tissue	*P*-value
**Tumor suppressor genes**			
*p16*			
Unmethylated	48 (67.6)	39 (86.7)	
Methylated	23 (32.4)	6 (13.3)	0.02
*DAPK*			
Unmethylated	50 (70.4)	40 (88.9)	
Methylated	21 (29.6)	5 (11.1)	0.02
*BRCA1*			
Unmethylated	58 (81.7)	41 (91.1)	
Methylated	13 (18.3)	4 (8.9)	0.16
*GSTP1*			
Unmethylated	49 (69)	38 (84.4)	
Methylated	22 (31)	7 (15.6)	0.06
*ECAD*			
Unmethylated	48 (67.6)	41 (91.1)	
Methylated	23 (32.4)	4 (8.9)	0.04*
*RASSF1*			
Unmethylated	35 (49.3)	35 (77.8)	
Methylated	36 (50.7)	10 (22.2)	0.02*
*MLH1*			
Unmethylated	67 (94.4)	44 (97.8)	
Methylated	4 (5.6)	1 (2.2)	0.38
**Tumor–specific loci**			
*MINT1*			
Unmethylated	40 (56.3)	39 (86.7)	
Methylated	31 (43.7)	6 (13.3)	0.01*
*MINT2*			
Unmethylated	34 (47.9)	40 (88.9)	
Methylated	37 (52.1)	5 (11.1)	<0.01*
*MINT31*			
Unmethylated	38 (53.5)	37 (82.2)	
Methylated	33 (46.5)	8 (17.8)	0.01*

Wilcoxon rank-sum test was used to calculate *P*-value

*Bonferroni correction of significance was applied

### Promoter methylation status in HPV positive (+) and HPV negative (-) HNSCC

**Fig 2:**
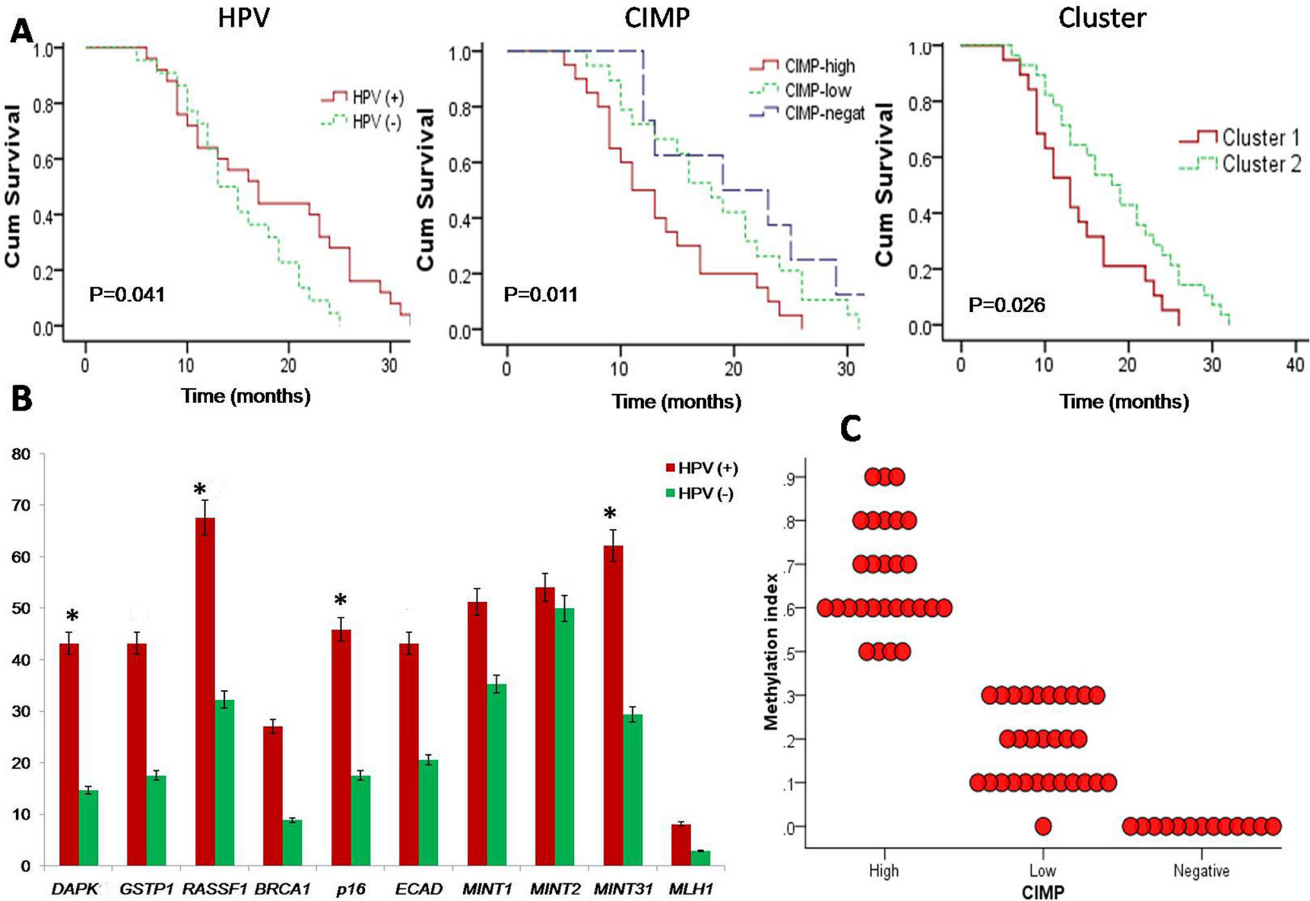
(A) Kaplan-Meier survival plots: (i) HPV (+) HNSCC tumors showing better survival compared to HPV (-) tumors. (ii) CIMP-high group of HNSCC tumors showing poorer survival compared to other. (iii) Two epigenetic cluster also showed differential survival with cluster-1 had a poor survival. (B) Frequency of promoter methylation of 10 tumor-related genes/loci in HPV (+) and HPV (-) HNSCC [*P<0.05 (Mantel–Cox Log–rank)]. (C) Methylation index (MI) in three CIMP-groups of HNSCC tumors.

In the study, HPV was detected in 37 out of 71 cases (52.11%) using consensus primers. The correlation between methylation of tumor-related genes and HPV was summarized in **Table 3**. Results shown that promoter methylation of *DAPK*, *RASSF1*, *p16* and *MINT31* were significantly higher in HPV positive (+) HNSCC patients compared to HPV negative (-) patients (*P* = 0.031, 0.013, 0.031 and 0.015) (**Fig 2B**). A highly significant association was found between HPV positive (+) tumors and CIMP-high group (*P* = 0.028). However, there was no correlation between CIMP-low and HPV (+) HNSCC (*P* = 0.477), when compared with HPV (-) HNSCC tumors.

**Table 3:** Frequency of DNA methylation of genes/loci and CIMP status analyzed in HPV (+) and HPV (-) HNSCC.

Gene/loci	HPV positive (%)	HPV negative (%)	*P*-value*
*DAPK*			
Methylated	16 (43.2)	5 (14.7)	
Unmethylated	21 (56.8)	29 (85.3)	0.031
*GSTP1*			
Methylated	16 (43.2)	6 (17.6)	
Unmethylated	21 (56.8)	28 (82.4)	0.057
*RASSF1*			
Methylated	25 (67.6)	11 (32.3)	
Unmethylated	12 (32.4)	23 (67.7)	0.013
*BRCA1*			
Methylated	10 (27.1)	3 (8.9)	
Unmethylated	27 (72.9)	31 (91.1)	0.066
*p16*			
Methylated	17 (45.9)	6 (17.6)	
Unmethylated	20 (54.1)	28 (82.4)	0.031
*ECAD*			
Methylated	16 (43.2)	7 (20.6)	
Unmethylated	21 (56.8)	27 (79.4)	0.087
*MINT1*			
Methylated	19 (51.3)	12 (35.3)	
Unmethylated	18 (48.7)	22 (64.7)	0.522
*MINT2*			
Methylated	20 (54.1)	17 (50)	
Unmethylated	17 (45.9)	17 (50)	0.782
*MINT31*			
Methylated	23 (62.2)	10 (29.4)	
Unmethylated	14 (37.8)	24 (70.6)	0.015
*MLH1*			
Methylated	3 (8.1)	1 (2.9)	
Unmethylated	34 (91.9)	33 (97.1)	0.327
CIMP status			
CIMP-negative	3 (8.1)	10 (29.4)	
CIMP-low	13 (35.1)	17 (50)	0.477
CIMP-high	21 (56.8)	7 (20.6)	0.028
*P*_trend_			0.001

* Chi square test used to calculate *P*-value and further adjusting for age, gender, smoking, betel-quid and tobacco chewing status

*P* < 0.05 considered as statistically significant

### CIMP in HNSCC tumor tissues

In this study, CIMP status was classified as CIMP-high (five or more methylated genes), CIMP-low (less than five methylated genes) and CIMP-negative (no methylated genes). Of the 71 HNSCC samples, 39.4% (28/71) were CIMP-high, 42.3% (30/71) were CIMP-low and 18.3% (13/71) were CIMP-negative.

### Correlation between environmental factors and CIMP

When we analyzed the data on environmental factors such as smoking, betel quid chewing and with CIMP and found smoking and tobacco chewing had strong correlation with CIMP-high (*P* = 0.008 and 0.034 respectively) compared to CIMP-negative. However, betel quid chewing had no significant correlation with CIMP-markers. We also had not found any significant differences between CIMP-low Vs CIMP-negative and CIMP-high Vs CIMP-low in terms of tobacco smoking or chewing (**Table 4**).

**Table 4:** Correlation between HPV, environmental and genetic characteristics and CIMP-status.

Characteristics	CIMP−H (N = 28)	CIMP−L (N = 30)	CIMP−N (N = 13)	*P*-values*		
				CIMP–H vs. CIMP−N	CIMP−L vs. CIMP−N	CIMP−H vs. CIMP-L
Smoking						
Yes	25 (89.3)	21 (70)	5 (38.5)			
No	3 (10.7)	9 (30)	8 (61.5)	0.008	0.044	0.214
Betel quid chewing						
Yes	20 (71.4)	20 (66.7)	7 (53.8)			
No	9 (28.6)	10 33.3)	6 (46.2)	0.303	0.249	0.968
Tobacco chewing						
Yes	25 (89.3)	22 (73.3)	6 (46.2)			
No	3 (10.7)	8 (26.7)	7 (53.8)	0.034	0.207	0.170
*GSTM1*						
Null	22 (78.6)	14 (46.7)	4 (30.8)			
Present	6 (21.4)	16 (53.3)	9 (69.2)	0.023	0.731	0.004
*GSTT1*						
Null	10 (35.7)	15 (50)	4 (30.8)			
Present	18 (64.3)	15 (50)	9 (69.2)	0.697	0.762	0.281
*CYP1A1* (T3801C)						
Wild (TT)	8 (28.6)	13 (43.3)	8 (61.5)			
Heterozygous (TC)	10 (35.7)	11 (36.7)	4 (30.8)	0.997	0.931	0.910
Variant (CC)	10 (35.7)	6 (20)	1 (7.7)	0.180	0.456	0.256
*XRCC1* (Arg399Gln)						
Arg/Arg	7 (25)	12 (40)	6 (46.2)			
Arg/Gln	14 (50)	11 (36.7)	6 (46.2)	0.108	0.624	0.281
Gln/Gin	7 (25)	7 (23.3)	1 (7.7)	0.056	0.230	0.434
*XRCC2* (Arg188His)						
Arg/Arg	14 (50)	22 (73.3)	10 (76.9)			
Arg/His	14 (50)	8 (26.7)	3 (23.1)	0.402	0.994	0.186

CIMP−H = CIMP-high; CIMP−L = CIMP-low and CIMP−N = CIMP-negative

* *P*-value was calculated by Chi-square test and further *P*-value was adjusting for age, gender, HPV, smoking, betel-quid and tobacco chewing status (as appropriate)

### Correlation between genetic factors and CIMP

We also correlated the genetic alteration data of carcinogen metabolizing (*GSTM1*, *GSTT1* and *CYP1A1*) and DNA repair (*XRCC1* and *XRCC2*) genes with CIMP panel data. The CIMP-high tumors had significantly higher frequency of *GSTM1* null, when compared to the CIMP-low and CIMP-negative tumors (*P* = 0.004 and 0.023 respectively). However, there was no significant correlation between *GSTT1* (null), *CYP1A1* (T3801C), *XRCC1* (Arg/Gln) and *XRCC2* (Arg/His) variant genotypes and different CIMP markers (**Table 4**).

### Survival correlates with CIMP and HPV

Survival was examined with respect to CIMP markers using Kaplan-Meier survival curves (**Fig 2A**). Analysis revealed that the overall median survival time of 47 patients out of 71 was 15 months [95% CI = 10.97–19.03], and the median survival time in CIMP-high, CIMP-low and CIMP-negative were 11 months [95% CI = 7.71 to 14.28], 18 months [95% CI = 13.73 to 22.26], and 19 months [95% CI = 5.14 to 32.85], respectively (*P*_*trend*_ = 0.011). Again median survival time for Cluster-1 and Cluster-2 characteristic were 13 and 18 months, respectively (*P* = 0.026) (**S1 Table**). The CIMP-high and Cluster-1 were significantly associated with poor survival in patients with HNSCC. Whereas, survival time for the HPV (+) HNSCC patients was better (17 months) than HPV (-) HNSCC patients (13 months) (*P* = 0.041).

### Identified tumor clusters and correlation with environmental, genetic and epigenetic characteristics

**Fig 3:**
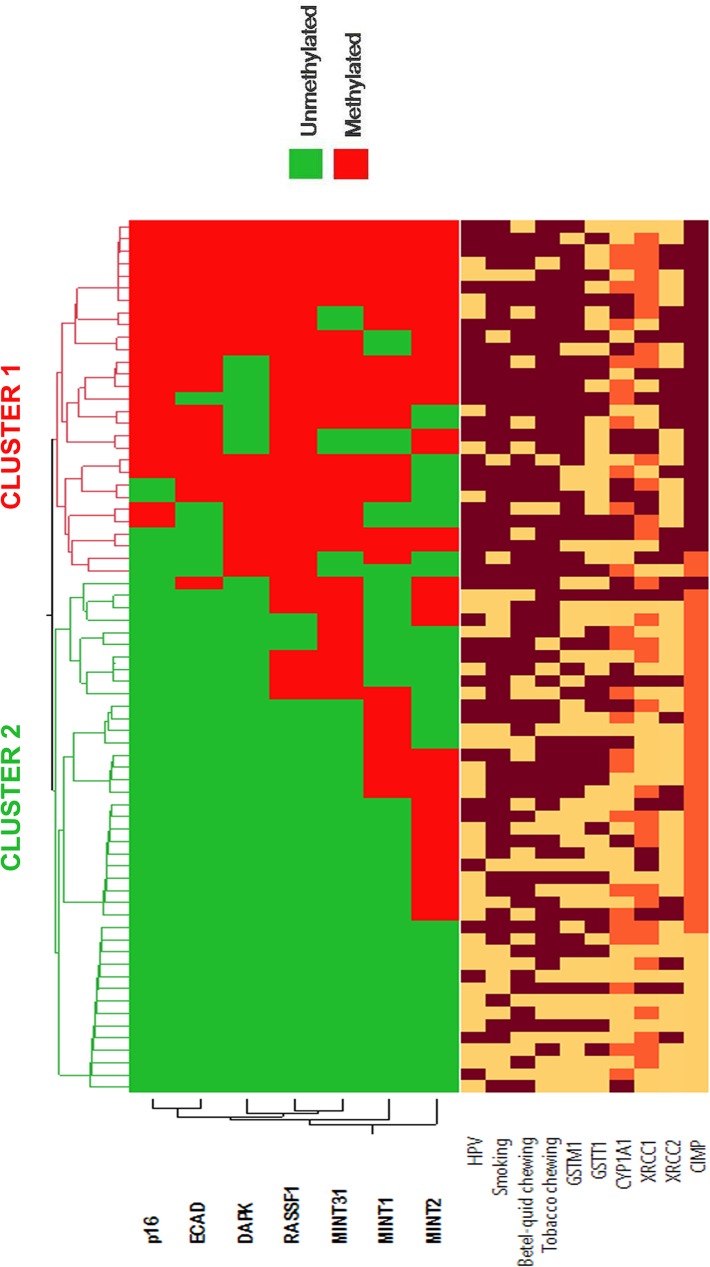
Unsupervised hierarchical clustering and heatmap was constructed based on promoter methylation data of HNSCC (using significantly methylated seven tumor-related genes/loci). The different factors in heatmap were represented by color variation: tobacco consumers, HPV presence and *GSTM1* null, *GSTT1* null (dark red color); tobacco non-consumers and HPV absence *GSTM1* present and *GSTT1* present (light yellow color). For *CYP1A1*, *XRCC1* and *XRCC2* status: wild type (dark red); heterozygous (orange red) and homozygous variant allele (light orange) and for CIMP status: CIMP-high (dark red), CIMP-low (orange red) and CIMP-negative (light color).

In the present study, we performed unsupervised hierarchical clustering and identified two classes or sub-groups based on promoter methylation data on tumor samples (**Fig 3**). We had constructed hierarchical clusters using seven genes/loci, as after adjustment seven gene/loci out of ten found to be significantly hypermethylated. The two identified clusters had distinct environmental, genetic and epigenetic features and are summarized in **Table 5**. The frequency of smoking (86.2%) and tobacco-chewing (89.7%), *GSTM1* null (82.8%) and *CYP1A1* (31.05%), *XRCC1* (27.6%) and *XRCC2* (48.3%) variant genotypes was higher in Cluster-1 compared to Cluster-2. However, only smoking, tobacco chewing and *GSTM1* null genotype had shown statistically significant variation among the clusters (*P* = 0.048, 0.034 and 0.002 respectively). Also, the frequency of HPV positive (+) HNSCC tumors was significantly higher (*P* = 0.009) in Cluster-1 compared to Cluster-2. CIMP-high group (93.1%) is significantly higher in of Cluster-1 (*P*<0.001) compared to Cluster-2, whereas, Cluster-2 characterized by CIMP-low (66.7%) and CIMP-negative groups (30.9%).

**Table 5:** Characteristics of the two identified clusters using hierarchical cluster analysis of promoter methylation data.

Characteristics	Cluster 1 (N = 29) (%)	Cluster 2 (N = 42) (%)	*P*-value
Smoking			
Yes	25 (86.2)	26 (61.9)	0.048
No	4 (13.8)	16 (38.1)	
Betel quid chewing			
Yes	21 (72.4)	26 (61.9)	0.360
No	8 (27.6)	16 (38.1)	
Tobacco chewing			
Yes	26 (89.7)	27 (64.3)	0.034
No	3 (10.3)	15 (35.7)	
*GSTM1*			
Null	24 (82.8)	16 (38.1)	0.002
Present	5 (17.2)	26 (61.9)	
*GSTT1*			
Null	11 (37.9)	18 (42.9)	0.474
Present	18 (62.1)	24 (57.1)	
*CYP1A1* (T3801)			
Wild (TT)	9 (31.05)	20(47.6)	
Heterozygous (TC)	11 (37.9)	14 (33.3)	0.997
Variant (CC)	9 (31.05)	8 (19.1)	0.371
*XRCC1* (Arg399Gln)			
Arg/Arg	8 (27.6)	17 (40.4)	
Arg/Gln	13 (44.8)	18 (42.9)	0.428
Gln/Gin	8 (27.6)	7 (16.7)	0.169
*XRCC2* (Arg188His)			
Arg/Arg	15 (51.7)	31 (73.8)	
Arg/His	14 (48.3)	11 (26.2)	0.481
His/His	0	0	
HPV status			
Present	22 (75.9)	15 (35.7)	0.009
Absent	7 (24.1)	27 (64.3)	
CIMP status			
Positive	27 (93.1)	1 (2.4)	
Low	2 (6.9)	28 (66.7)	<0.001
Negative	0	13 (30.9)	<0.001
*P*_*trend*_			<0.001

*P*-values were adjusted for age, gender, smoking, HPV, betel-quid and tobacco chewing as appropriate

## Discussion

The development and progression of HNSCC is a multi-step process modulated by genetic, epigenetic and environmental factors [2, 44, 45]. The major environmental factors such as tobacco smoking and chewing as well as HPV infection may lead to a wide range of genetic and epigenetic proceedings that promote genomic instability and endorse tumor development [3, 5, 9]. Promoter hypermethylation profile of tumor-related genes was anticipated to be crucial and frequent in different cancers. Many epigenetic events in carcinogenic pathways have been studied recently and revealed the methods for detecting CpG island promoter methylation pattern to stratify high risk groups among different cancers [15, 17, 18, 34]. This helps in detection of early onset of cancer, and predicts clinical outcomes. Therefore, it is indispensable to study the methylation status of a panel of representative genes in HNSCC. Here, in this study, we analyzed the aberrant promoter methylation profile of HNSCC using seven important tumor-related pathway genes (*p16*, *DAPK*, *GSTP1*, *ECAD*, *RASSF1*, *MLH1* and *BRCA1*) and three methylated loci (*MINT1*, *MINT2* and *MINT31*) in the high cancer incidence zone of Northeast India. In our study, we also correlate aberrant methylation status of patients with genetic (polymorphisms of *GSTM1*, *GSTT1*, *CYP1A1*, *XRCC1* and *XRCC2*) and environmental factor (smoking, betel quid and tobacco chewing) as well as with survival data.

We found a significantly high level of *p16*, *DAPK*, *ECAD*, *RASSF1*, *MINT1*, *MINT2* and *MINT31* hypermethylation in HNSCC tissues compared to normal tissue samples, reflecting the possible involvement of epigenetic alteration toward the development and progression of HNSCC. Our present investigation had covered a broad group of tumor-related pathway genes, including *p16* (cell-cycle control), *DAPK*, *RASSF1* (apoptosis), *BRCA1*, *MLH1* (DNA repair), *ECAD* (cell-cell adhesion), *GSTP1* (carcinogen metabolism), *MINT1*, *MINT2* and *MINT31* (methylated loci in tumors).

Many previous epigenetic studies elucidated existence of HPV mediated DNA-methylation in HNSCC [46, 47]. Recent study from the Northeast India using a panel of 10 genes, explained the role of HPV and tobacco in the genesis of UADT cancer [32]. However, we further analyzed hypermethylation of individual genes/loci separately in HPV (+) and HPV (-) cases. We found promoter methylation of *DAPK*, *p*16, *RASSF1* and *MINT31* was significantly associated with HPV (+) tumors of HNSCC. Therefore, HPV appeared to be a causative agent for alterations of CpG island methylation of tumor-suppressive genes in HNSCC. In general, HPV (+) HNSCC is associated with a more favorable survival [9], our study also explored HPV (+) patients of HNSCC had better survival rate compared to HPV (-) patients reflecting a possible role in prognosis.

In many cancer types, especially in colorectal cancer, CpG Island Methylator Phenotype (CIMP) was used to identify clinically and pathologically relevant subsets of tumors. Toyota et al. introduced CpG island methylator phenotype (CIMP) to classify cancer based on the methylation status of a panel of genes [21]. In colorectal cancer, CIMP tumors had distinct epidemiologic characteristics, microsatellite instability (MSI) profile BRAF mutations and survival, compared to non-CIMP tumors [48, 49]. CIMPs have been reported for a number of cancers, including upper aerodigestive tract (UADT) [32], oral cancer [50], and oesophageal squamous cell carcinoma [51] and breast cancer [19]. However, only single study was there in HNSCC, which explain CIMP in the sub-group of HPV (+) tumor of HNSCC [33]. The hypermethylation profile of gene promoters is diverse for each type of cancers and also the detection method and multiple gene selection for CIMP-panel varies among the studies. Based on the hypermethylation pattern of 10 tumor-related genes/loci, we classified HNSCC in three groups: CIMP-high, CIMP-low and CIMP-negative. We observed distinct characteristics of tumor within the CIMP-high and CIMP-negative groups. Frequencies of smoking, tobacco chewing and *GSTM1* null genotypes of patients were significantly higher in CIMP-high groups compared to CIMP-negative. Genetic and environmental characteristics may direct to CIMP characteristics of HNSCC tumors. We also observed an inclination poor survival rate in CIMP-high tumors compared to CIMP-negative group; indicate CIMP-high may be a predictor of a poor prognosis of HNSCC in Northeast Indian population. It is perhaps not unexpected that we have found correlations between the CIMP-high and patient poor outcome, if we compared our data on the CIMP and survival in HNSCC with other previously described cancers [52, 53]. A profound understanding of CIMP could allow designing of better treatment strategies for HNSCC.

Epigenetic clustering of HNSCC patients was done based on DNA methylation profiles in tumor tissue samples. Hierarchical cluster analysis identified two distinct subsets, one subset of HNSCC patients with high frequency of smoking, tobacco chewing habits, *GSTM1* null and *CYP1A1* (CC) variant genotype. The cluster-1 also characterized by HPV (+) cases, and contained CIMP-high group, reflecting a possible correlation between DNA methylation and genetic and environmental factors in HPV associated HNSCC. Thus, epigenetic and along with genetic alteration with a combination of environmental factors may also play a role in a subset of HNSCC. In our previous study, we found strong interaction between carcinogen metabolizing genes (*GSTM1* and *GSTT1*) and environmental factors in HNSCC [2]. The interaction of Phase-I (*CYP1A1*) and Phase-II (*GSTM1* & *GSTT1*) tobacco carcinogens metabolizing genes may elucidate the accumulation of the larger amount of toxic substances inside the body that might play the major part during the development of HNSCC. Also, one of our other study explored the interaction between tobacco and DNA repair (*XRCC1* and *XRCC2*) gene polymorphisms and cross talk between these two DNA repair genes towards susceptibility of HNSCC [37]. Recent study by Tahara et al. [39] found association between *GSTM1* null genotype and increased susceptibility to CpG hypermethylation, however they found reduced susceptibility to CpG hypermethylation of *DAPK* gene with *XRCC1* codon 399 Gln ⁄ Gln genotype and concluded that *GSTM1* null genotype may have a role in CpG hypermethylation related gastric carcinogenesis. We also found significant association between promoter hypermethylation of CpG island and *GSTM1* null genotype in HNSCC. Hierarchical cluster analysis also confirmed this association and thus indicated a possible role of carcinogen metabolizing genes in promoter hypermethylation of tumor-related genes.

In conclusion, promoter hypermethylation of tumor-related genes/loci plays an important role to the development and progression of cancers. Our investigation revealed that epigenetic alteration play as a major mechanism in the carcinogenesis of HNSCC. We also identified distinct subsets of HNSCC based on differential epigenetic, genetic, HPV and environmental characteristics. Furthermore, we could assess the outcome of patients, based on HPV, CIMP and cluster (promoter methylation profiles) characteristic. To the best of our knowledge, this is the first epigenetic, genetic, environmental and prognostic study conducted on HNSCC patients from India. On the whole, this type of study based on promoter methylation analysis in combination with genetic and environmental factors would be helpful in identification of diagnostic and prognostic biomarkers and therapy. Further studies on other population and with larger sample sizes are required for functional validation before the clinical inference can be considered.

## Supporting Information

S1 Table Survival data of HNSCC patients. (DOC)

S2 Table List of primers used in the study. (DOC)

## References

[1] Jemal A, Bray F, Center MM, Ferlay J, Ward E, Forman D. Global cancer statistics. CA: a cancer journal for clinicians. 2011;61(2):69–90. Epub 2011/02/08. 10.3322/caac.20107 . 21296855

[2] Choudhury JH, Ghosh SK. Gene-environment interaction and susceptibility in head and neck cancer patients and in their first-degree relatives: a study of Northeast Indian population. Journal of oral pathology & medicine: official publication of the International Association of Oral Pathologists and the American Academy of Oral Pathology. 2014. Epub 2014/08/30. 10.1111/jop.12249 . 25167831

[3] Fotopoulos G, Pavlidis N. The role of human papilloma virus and p16 in occult primary of the head and neck: A comprehensive review of the literature. Oral oncology. 2014. Epub 2014/12/04. 10.1016/j.oraloncology.2014.10.018 . 25467774

[4] Ramshankar V, Krishnamurthy A. Human papilloma virus in head and neck cancers-role and relevance in clinical management. Indian journal of surgical oncology. 2013;4(1):59–66. Epub 2014/01/16. 10.1007/s13193-012-0196-5 24426701 PMC3578549

[5] Basu R, Mandal S, Ghosh A, Poddar TK. Role of tobacco in the development of head and neck squamous cell carcinoma in an eastern Indian population. Asian Pacific journal of cancer prevention: APJCP. 2008;9(3):381–6. Epub 2008/11/08. . 18990006

[6] Anuradha V, Anand BB, Suresh AV, Sinha S, Babu SC, Suresh K. Palliative chemotherapy in head and neck squamous cell cancer—What is best in Indian population? A time without symptoms, treatment toxicity score based study. Indian journal of medical and paediatric oncology: official journal of Indian Society of Medical & Paediatric Oncology. 2013;34(1):11–5. Epub 2013/07/24. 10.4103/0971-5851.113404 23878480 PMC3715971

[7] Fakhry C, Westra WH, Li S, Cmelak A, Ridge JA, Pinto H, et al Improved survival of patients with human papillomavirus-positive head and neck squamous cell carcinoma in a prospective clinical trial. Journal of the National Cancer Institute. 2008;100(4):261–9. Epub 2008/02/14. 10.1093/jnci/djn011 . 18270337

[8] Ho T, Wei Q, Sturgis EM. Epidemiology of carcinogen metabolism genes and risk of squamous cell carcinoma of the head and neck. Head & neck. 2007;29(7):682–99. Epub 2007/02/03. 10.1002/hed.20570 . 17274053

[9] Perez-Ordonez B, Beauchemin M, Jordan RC. Molecular biology of squamous cell carcinoma of the head and neck. Journal of clinical pathology. 2006;59(5):445–53. Epub 2006/04/29. 10.1136/jcp.2003.007641 16644882 PMC1860277

[10] Lothaire P, de Azambuja E, Dequanter D, Lalami Y, Sotiriou C, Andry G, et al Molecular markers of head and neck squamous cell carcinoma: promising signs in need of prospective evaluation. Head & neck. 2006;28(3):256–69. Epub 2005/11/15. 10.1002/hed.20326 . 16284973

[11] Lacko M, Oude Ophuis MB, Peters WH, Manni JJ. Genetic polymorphisms of smoking-related carcinogen detoxifying enzymes and head and neck cancer susceptibility. Anticancer research. 2009;29(2):753–61. Epub 2009/04/01. . 19331232

[12] Hansen KD, Timp W, Bravo HC, Sabunciyan S, Langmead B, McDonald OG, et al Increased methylation variation in epigenetic domains across cancer types. Nature genetics. 2011;43(8):768–75. Epub 2011/06/28. 10.1038/ng.865 21706001 PMC3145050

[13] Rodriguez-Paredes M, Esteller M. Cancer epigenetics reaches mainstream oncology. Nature medicine. 2011;17(3):330–9. Epub 2011/03/10. 10.1038/nm.2305 . 21386836

[14] Kanai Y, Hirohashi S. Alterations of DNA methylation associated with abnormalities of DNA methyltransferases in human cancers during transition from a precancerous to a malignant state. Carcinogenesis. 2007;28(12):2434–42. Epub 2007/09/26. 10.1093/carcin/bgm206 . 17893234

[15] Teodoridis JM, Strathdee G, Brown R. Epigenetic silencing mediated by CpG island methylation: potential as a therapeutic target and as a biomarker. Drug resistance updates: reviews and commentaries in antimicrobial and anticancer chemotherapy. 2004;7(4–5):267–78. Epub 2004/11/10. 10.1016/j.drup.2004.06.005 . 15533764

[16] Baylin SB, Esteller M, Rountree MR, Bachman KE, Schuebel K, Herman JG. Aberrant patterns of DNA methylation, chromatin formation and gene expression in cancer. Human molecular genetics. 2001;10(7):687–92. Epub 2001/03/21. . 11257100 10.1093/hmg/10.7.687

[17] MAFG mediates CIMP in BRAF-mutant colorectal cancer. Cancer discovery. 2014;4(11):OF11 Epub 2014/11/05. 10.1158/2159-8290.CD-RW2014-200 . 25367952

[18] Laskar RS, Ghosh SK, Talukdar FR. Rectal cancer profiling identifies distinct subtypes in India based on age at onset, genetic, epigenetic and clinicopathological characteristics. Molecular carcinogenesis. 2014. Epub 2014/11/25. 10.1002/mc.22250 . 25418895

[19] Roessler J, Ammerpohl O, Gutwein J, Steinemann D, Schlegelberger B, Weyer V, et al. The CpG island methylator phenotype in breast cancer is associated with the lobular subtype. Epigenomics. 2014:1–13. Epub 2014/10/28. 10.2217/epi.14.74 . 25347269

[20] Shaw RJ, Hall GL, Lowe D, Bowers NL, Liloglou T, Field JK, et al CpG island methylation phenotype (CIMP) in oral cancer: associated with a marked inflammatory response and less aggressive tumour biology. Oral oncology. 2007;43(9):878–86. Epub 2007/01/30. 10.1016/j.oraloncology.2006.10.006 . 17257884

[21] Toyota M, Ahuja N, Ohe-Toyota M, Herman JG, Baylin SB, Issa JP. CpG island methylator phenotype in colorectal cancer. Proceedings of the National Academy of Sciences of the United States of America. 1999;96(15):8681–6. Epub 1999/07/21. 10411935 10.1073/pnas.96.15.8681 PMC17576

[22] Hasegawa M, Nelson HH, Peters E, Ringstrom E, Posner M, Kelsey KT. Patterns of gene promoter methylation in squamous cell cancer of the head and neck. Oncogene. 2002;21(27):4231–6. Epub 2002/06/26. 10.1038/sj.onc.1205528 . 12082610

[23] Viswanathan M, Tsuchida N, Shanmugam G. Promoter hypermethylation profile of tumor-associated genes p16, p15, hMLH1, MGMT and E-cadherin in oral squamous cell carcinoma. International journal of cancer Journal international du cancer. 2003;105(1):41–6. Epub 2003/04/03. 10.1002/ijc.11028 . 12672028

[24] Steinmann K, Sandner A, Schagdarsurengin U, Dammann RH. Frequent promoter hypermethylation of tumor-related genes in head and neck squamous cell carcinoma. Oncology reports. 2009;22(6):1519–26. Epub 2009/11/04. . 19885608 10.3892/or_00000596

[25] Pierini S, Jordanov SH, Mitkova AV, Chalakov IJ, Melnicharov MB, Kunev KV, et al Promoter hypermethylation of CDKN2A, MGMT, MLH1, and DAPK genes in laryngeal squamous cell carcinoma and their associations with clinical profiles of the patients. Head & neck. 2014;36(8):1103–8. Epub 2013/06/28. 10.1002/hed.23413 . 23804521

[26] Roh JL, Wang XV, Manola J, Sidransky D, Forastiere AA, Koch WM. Clinical correlates of promoter hypermethylation of four target genes in head and neck cancer: a cooperative group correlative study. Clinical cancer research: an official journal of the American Association for Cancer Research. 2013;19(9):2528–40. Epub 2013/02/28. 10.1158/1078-0432.CCR-12-3047 23444219 PMC3642232

[27] Park SJ, Rashid A, Lee JH, Kim SG, Hamilton SR, Wu TT. Frequent CpG island methylation in serrated adenomas of the colorectum. The American journal of pathology. 2003;162(3):815–22. Epub 2003/02/25. 10.1016/S0002-9440(10)63878-3 12598316 PMC1868094

[28] Righini CA, de Fraipont F, Timsit JF, Faure C, Brambilla E, Reyt E, et al Tumor-specific methylation in saliva: a promising biomarker for early detection of head and neck cancer recurrence. Clinical cancer research: an official journal of the American Association for Cancer Research. 2007;13(4):1179–85. Epub 2007/02/24. 10.1158/1078-0432.CCR-06-2027 . 17317827

[29] Ogi K, Toyota M, Ohe-Toyota M, Tanaka N, Noguchi M, Sonoda T, et al Aberrant methylation of multiple genes and clinicopathological features in oral squamous cell carcinoma. Clinical cancer research: an official journal of the American Association for Cancer Research. 2002;8(10):3164–71. Epub 2002/10/11. . 12374684

[30] Supic G, Kozomara R, Jovic N, Zeljic K, Magic Z. Prognostic significance of tumor-related genes hypermethylation detected in cancer-free surgical margins of oral squamous cell carcinomas. Oral oncology. 2011;47(8):702–8. Epub 2011/06/24. 10.1016/j.oraloncology.2011.05.014 . 21697000

[31] Su PF, Huang WL, Wu HT, Wu CH, Liu TY, Kao SY. p16(INK4A) promoter hypermethylation is associated with invasiveness and prognosis of oral squamous cell carcinoma in an age-dependent manner. Oral oncology. 2010;46(10):734–9. Epub 2010/08/24. 10.1016/j.oraloncology.2010.07.002 . 20729138

[32] Talukdar FR, Ghosh SK, Laskar RS, Kannan R, Choudhury B, Bhowmik A. Epigenetic pathogenesis of human papillomavirus in upper aerodigestive tract cancers. Molecular carcinogenesis. 2014. Epub 2014/09/13. 10.1002/mc.22214 . 25213493

[33] Lechner M, Fenton T, West J, Wilson G, Feber A, Henderson S, et al Identification and functional validation of HPV-mediated hypermethylation in head and neck squamous cell carcinoma. Genome medicine. 2013;5(2):15 Epub 2013/02/20. 10.1186/gm419 23419152 PMC3706778

[34] Dong Y, Zhao H, Li H, Li X, Yang S. DNA methylation as an early diagnostic marker of cancer (Review). Biomedical reports. 2014;2(3):326–30. Epub 2014/04/22. 10.3892/br.2014.237 24748968 PMC3990206

[35] Wise-Draper TM, Wells SI. Papillomavirus E6 and E7 proteins and their cellular targets. Frontiers in bioscience: a journal and virtual library. 2008;13:1003–17. Epub 2007/11/06. . 17981607 10.2741/2739

[36] Hernandez JM, Siegel EM, Riggs B, Eschrich S, Elahi A, Qu X, et al DNA methylation profiling across the spectrum of HPV-associated anal squamous neoplasia. PloS one. 2012;7(11):e50533 Epub 2012/12/12. 10.1371/journal.pone.0050533 23226306 PMC3511539

[37] Choudhury JH, Choudhury B, Kundu S, Ghosh SK. Combined effect of tobacco and DNA repair genes polymorphisms of XRCC1 and XRCC2 influence high risk of head and neck squamous cell carcinoma in northeast Indian population. Med Oncol. 2014;31(8):67 Epub 2014/06/25. 10.1007/s12032-014-0067-8 . 24958516

[38] Choudhury JH, Singh SA, Kundu S, Choudhury B, Talukdar FR, Srivasta S, et al. Tobacco carcinogen-metabolizing genes CYP1A1, GSTM1, and GSTT1 polymorphisms and their interaction with tobacco exposure influence the risk of head and neck cancer in Northeast Indian population. Tumour biology: the journal of the International Society for Oncodevelopmental Biology and Medicine. 2015. Epub 2015/03/01. 10.1007/s13277-015-3246-0 . 25724184

[39] Tahara T, Shibata T, Nakamura M, Okubo M, Yamashita H, Yoshioka D, et al Polymorphisms of DNA repair and xenobiotic genes predispose to CpG island methylation in non-neoplastic gastric mucosa. Helicobacter. 2011;16(2):99–106. Epub 2011/03/26. 10.1111/j.1523-5378.2011.00821.x . 21435086

[40] Mondal R, Ghosh SK, Choudhury JH, Seram A, Sinha K, Hussain M, et al Mitochondrial DNA copy number and risk of oral cancer: a report from Northeast India. PloS one. 2013;8(3):e57771 Epub 2013/03/08. 10.1371/journal.pone.0057771 23469236 PMC3587625

[41] Chen J, Cheng M, Yi L, Jiang CB. Relationship between CYP1A1 genetic polymorphisms and renal cancer in China. Asian Pacific journal of cancer prevention: APJCP. 2011;12(9):2163–6. Epub 2012/02/03. . 22296350

[42] Nazemalhosseini Mojarad E, Kuppen PJ, Aghdaei HA, Zali MR. The CpG island methylator phenotype (CIMP) in colorectal cancer. Gastroenterology and hepatology from bed to bench. 2013;6(3):120–8. Epub 2014/05/17. 24834258 PMC4017514

[43] Mani S, Szymanska K, Cuenin C, Zaridze D, Balassiano K, Lima SC, et al DNA methylation changes associated with risk factors in tumors of the upper aerodigestive tract. Epigenetics: official journal of the DNA Methylation Society. 2012;7(3):270–7. Epub 2012/03/21. 10.4161/epi.7.3.19306 22430803 PMC3335950

[44] Arantes LM, de Carvalho AC, Melendez ME, Carvalho AL, Goloni-Bertollo EM. Methylation as a biomarker for head and neck cancer. Oral oncology. 2014;50(6):587–92. Epub 2014/03/25. 10.1016/j.oraloncology.2014.02.015 . 24656975

[45] Bernstein JM, Bernstein CR, West CM, Homer JJ. Molecular and cellular processes underlying the hallmarks of head and neck cancer. Eur Arch Otorhinolaryngol. 2013;270(10):2585–93. Epub 2012/12/25. 10.1007/s00405-012-2323-x . 23263268

[46] Lleras RA, Smith RV, Adrien LR, Schlecht NF, Burk RD, Harris TM, et al Unique DNA methylation loci distinguish anatomic site and HPV status in head and neck squamous cell carcinoma. Clinical cancer research: an official journal of the American Association for Cancer Research. 2013;19(19):5444–55. Epub 2013/07/31. 10.1158/1078-0432.CCR-12-3280 23894057 PMC3892374

[47] Worsham MJ, Chen KM, Ghanem T, Stephen JK, Divine G. Epigenetic modulation of signal transduction pathways in HPV-associated HNSCC. Otolaryngology—head and neck surgery: official journal of American Academy of Otolaryngology-Head and Neck Surgery. 2013;149(3):409–16. Epub 2013/06/06. 10.1177/0194599813490895 23736812 PMC3935612

[48] Ogino S, Nosho K, Kirkner GJ, Kawasaki T, Meyerhardt JA, Loda M, et al CpG island methylator phenotype, microsatellite instability, BRAF mutation and clinical outcome in colon cancer. Gut. 2009;58(1):90–6. Epub 2008/10/04. 10.1136/gut.2008.155473 18832519 PMC2679586

[49] Lochhead P, Kuchiba A, Imamura Y, Liao X, Yamauchi M, Nishihara R, et al Microsatellite instability and BRAF mutation testing in colorectal cancer prognostication. Journal of the National Cancer Institute. 2013;105(15):1151–6. Epub 2013/07/24. 10.1093/jnci/djt173 23878352 PMC3735463

[50] Jithesh PV, Risk JM, Schache AG, Dhanda J, Lane B, Liloglou T, et al The epigenetic landscape of oral squamous cell carcinoma. British journal of cancer. 2013;108(2):370–9. Epub 2013/01/05. 10.1038/bjc.2012.568 23287992 PMC3566828

[51] Ling Y, Huang G, Fan L, Wei L, Zhu J, Liu Y, et al CpG island methylator phenotype of cell-cycle regulators associated with TNM stage and poor prognosis in patients with oesophageal squamous cell carcinoma. Journal of clinical pathology. 2011;64(3):246–51. Epub 2010/12/21. 10.1136/jcp.2010.082875 . 21169275

[52] Li X, Hu F, Wang Y, Yao X, Zhang Z, Wang F, et al CpG island methylator phenotype and prognosis of colorectal cancer in Northeast China. BioMed research international. 2014;2014:236361 Epub 2014/09/23. 10.1155/2014/236361 25243122 PMC4163374

[53] Kang KJ, Min BH, Ryu KJ, Kim KM, Chang DK, Kim JJ, et al. The Role of the CpG Island Methylator Phenotype on Survival Outcome in Colon Cancer. Gut and liver. 2014. Epub 2014/08/30. 10.5009/gnl13352 . 25167802 PMC4351027

