# Supplementary material for: Promoter Hypermethylation Profiling Identifies Subtypes of Head and Neck Cancer with Distinct Viral, Environmental, Genetic and Survival Characteristics
Source: PLoS One. 2015 Jun 22;10(6):e0129808. doi: 10.1371/journal.pone.0129808 (PMC4476679; doi:10.1371/journal.pone.0129808)
Supplement: S1 Table — (DOC) [file pone.0129808.s001.doc]

**Table S1:**  Survival data of HNSCC patients

| Variables | Median (months) | 95% Confidence Interval | | *P*-value |
| --- | --- | --- | --- | --- |
|  |  | Lower | Upper |  |
| Overall | 15 | 10.97 | 19.03 |  |
| Cluster-1 | 13 | 9.83 | 16.16 | 0.026 |
| Cluster-2 | 18 | 13.85 | 22.14 |  |
| CIMP-High | 11 | 7.71 | 14.28 | 0.011* |
| CIMP-Low | 18 | 13.73 | 22.26 |  |
| CIMP-Negative | 19 | 5.14 | 32.85 |  |
| HPV (-) | 13 | 10.24 | 15.75 | 0.041 |
| HPV (+) | 17 | 12.13 | 21.86 |  |

**P trend*
