# Supplementary material for: Promoter Hypermethylation Profiling Identifies Subtypes of Head and Neck Cancer with Distinct Viral, Environmental, Genetic and Survival Characteristics
Source: PLoS One. 2015 Jun 22;10(6):e0129808. doi: 10.1371/journal.pone.0129808 (PMC4476679; doi:10.1371/journal.pone.0129808)
Supplement: S2 Table — (DOC) [file pone.0129808.s002.doc]

| **Primer name** | **Primer sequence (5’-3’)** | **Gene/loci** |
| --- | --- | --- |
| **HPV My09-F HPV My11-R** | CGT CCM ARR GGA WAC TGA TC  GCM CAG GGW CAT AAY AAT GG | HPV L1 |
| **GSTM1-F**  **GSTM1-R** | GAACTCCCTGAAAAGCTAAAGC  GTTGGGCTCAAATATACGGTGG | GSTM1 |
| **GSTT1-F**  **GSTT1-R** | TTCCTTACTGGTCCTCACATTCTC  TCACGGGATCATGGCCAGCA | GSTT1 |
| **CYP1A1-F**  **CYP1A1-R** | ACTGCCACTTCAGCTGTCT  GCTGCATTTGGAAGTGCTC | CYP1A1 |
| **XRCC1-F**  **XRCC1-R** | TTGTGCTTTCTCTGTGTCCA  TCCTCCAGCCTTTTCTGATA | XRCC1 |
| **XRCC2-F**  **XRCC2-R** | TCACCCATCTCTCTGCCTTTTG  TTCTGATGAGCTCGAGGCTTTC | XRCC2 |
| **P16-MF**  **P16-MR** | TTA TTA GAG GGT GGG GCG GATCGC  GAC CCC GAA CCG CGA CCG TAA | *P16* |
| **P16-UF**  **P16-UR** | TTA TTA GAG GGT GGG GTG GATTGT  CAA CCC CAA ACC ACA ACC ATA A |
| **MLH1 MF**  **MLH1 MR** | GATAGC GAT TTT TAA CGC  TCT ATA AAT TAC TAA ATC TCT TCG | *MLH1* |
| **MLH1 UF**  **MLH1 UR** | AGAGTG GAT AGT GAT TTT TAA TGT  ACT CTA TAA ATT ACT AAA TCT CTT CA |
| **MINT1 MF**  **MINT1 MR** | AAT TTT TTT ATA TAT ATT TTC GAA GC  AAA AAC CTC AAC CCC GCG | *MINT1* |
| **MINT1 UF**  **MINT1 UR** | AAT TTT TTT ATA TAT ATT TTT GAA GTG T  AAC AAA AAA CCT CAA CCC CAC A |
| **MINT2 MF**  **MINT2 MR** | TTG TTA AAG TGT TGAG TTC GTC  AAT AAC GAC GAT TCC GTA CG | *MINT2* |
| **MINT2 UF**  **MINT2 UR** | GAT TTT GTT AAA GTG TTG AGT TTG TT  CAA AAT AAT AAC AAC AAT TCC ATA CA |
| **MINT31 MF**  **MINT31 MR** | TGT TGG GGA AGT GTT TTT CGG C  CGA AAA CGA AAC GCC GCG | *MINT31* |
| **MINT31 UF**  **MINT31 UR** | TAG ATG TTG GGG AAG TGT TTT TTG GT  TAA ATA CCC AAA AAC AAA ACA CCA CA |
| **ECAD-MF ECAD-MR** | TTA GGT TAG AGG GTT ATC GCG T TAA CTA AAA ATT CAC CTA CCG AC | *E-Cadherin* |
| **ECAD-UF ECAD-UR** | TAA TTT TAG GTT AGA GGG TTA TTG T CAC AAC CAA TCA ACA ACA CA |
| **DAPK-MF DAPK-MR** | GGA TAG TCG GAT CGA GTT AAC GTC CCC TCC CAA ACG CCG A | *DAPK* |
|
| **DAPK-UF DAPK-UR** | GGA GGA TAG TTG GAT TGA GTT AAT GTT CAA ATC CCT CCC AAA CAC CAA |
| **RASSF1-MF RASSF1-MR** | TTT TTC CAT TTC GCG TCT CT CGT TTT TGC CCT TTC TTC GC | *RASSF1* |
| **RASSF1-UF RASSF1-UR** | TCACCCATTTTTCCATTTCTCT CTT TTT TTC CCT TTC TTC TCT T |
| **GSTP1-MF GSTP1-MR** | GAA GTT GGC GAG GTC GAG TTT C ACC CGC CAC AAC CCG AAA AAA CG | *GSTP1* |
| **GSTP1-UF GSTP1-UR** | GGG AAG TTG GTG AGG TTG AGT TTT CAA CCC ACC ACA ACC CAA AAA ACA |

**Table S2:** List of primers used in the study
